# Identification and analysis of ship waiting behavior outside the port based on AIS data

**DOI:** 10.1038/s41598-023-38080-2

**Published:** 2023-07-12

**Authors:** Jianwen Ma, Yue Zhou, Zhaoxin Zhu

**Affiliations:** 1 International Business School, Shandong Jiaotong University, Weihai, 264200 China; 2 College of Merchant Marine, Shanghai Maritime University, Shanghai, 201306 China; 3 School of Navigation and Shipping, Shandong Jiaotong University, Weihai, 264200 China

**Keywords:** Engineering, Mathematics and computing

## Abstract

Port congestion caused by ship detention is an important reason for the obstruction of the shipping supply chain. In this paper, a method of excavating the ship waiting behavior outside the port is proposed based on the automatic identification system (AIS) data and considering uncontrollable factors. Firstly, combined with the analysis of ship navigation behavior, the speed threshold of the ship waiting outside the port is defined through average speed. Secondly, the range of ships berthing in the port is distinguished, and the different waiting behavior of ships are clarified. Finally, the situation of different types and sizes of ships waiting outside the port is analyzed. The paper found that ships of different types and sizes have distinctive clustered waiting behavior, and there are significant differences in waiting time. At the ship type, bulk cargo ship have the highest number among ships waiting outside the port, followed by tanker and container ship; at the ship scale, basically, the number of ships waiting outside the port decreases with the increase of the scale, but the average waiting time is longer. It has an excellent practical promotion value for the application of AIS data and development of shipping.

## Introduction

Ports are hubs of the global logistics supply chain and nodes of shipping. A smooth and efficient port is the key to ensuring the orderly operation of the logistics supply chain and the basis for ensuring maritime safety. Due to multiple factors, such as international trade relations and coronavirus disease 2019 (COVID-19), the congestion problem in ports is becoming increasingly severe. The number of ships waiting outside the port is increasing, and the waiting time is rising sharply, resulting in a sharp drop in the turnover efficiency of ships and an increase in the hidden danger of navigation in the port waters^1–3^. Ship waiting behavior outside the port refers to the behavior that ships scheduled to enter the port cannot dock at the berth due to port congestion and need to anchor or moor near the port waters to wait for entry^4^. In order to optimize port traffic organization and ship route planning, it is particularly important to excavate the ship waiting behavior outside the port and accurately identify the ship waiting in the port waters.

At present, AIS equipped by the ship can display dynamic navigation information such as sailing and anchoring. However, since these data are manually input, there will be problems such as wrong input and missing input, which is unsuitable for relevant research and analysis. It is a research hotspot in the field of transportation engineering to identify and analyze various ship behavior from AIS data^5–15^. There are also many achievements in the research on ship behavior recognition in ports and nearby waters. Wang et al. put forward a ship anchoring identification model based on ship driving behavior, analyzed the difference between ship anchoring and berthing time, and reflected the impact of COVID-19^16^; Huang et al. proposed a feature-driven recognition and automatic classification method for the two kinds of stopping behavior of ships: berthing and anchoring^17^; Zheng et al. analyzed the abnormal stay behavior of ships in port waters based on the characteristics of ship historical track data^18^; Wang et al. proposed an assessment method to quantify the impact of COVID-19 on ship behavior based on the AIS data of Oslo port, Norway, and analyzed the arrival time and berthing time of ships under the influence of COVID-19 by using the improved density-based spatial clustering of applications with noise (DBSCAN) algorithm^19^; Zheng et al. proposed a method for extracting the ship stay track based on multi-constraint conditions, which mined the stay area of the port berth by setting up multiple thresholds^20^; Liu et al. put forward a method based on clustering algorithm to identify ship anchoring behavior and excavate anchoring area in view of the situation that it is difficult to identify and supervise the coastal ships anchoring at sea^21^. Other scholars have analyzed and studied the congestion level of ports based on AIS data. AbuAlhaol et al. excavated three indicators of spatial complexity, spatial density, and time-critical point to evaluate the port congestion level from the AIS data, which are used to predict the future congestion of the port^22^; Peng et al. used the DBSCAN method and convex hull theory to identify berths and anchorage areas of the top 20 container ports in the world, and proposed a method to describe port congestion according to traffic volume and ship turnover time^23^.

Although the above research results have been used to excavate and identify the stay behavior in the port waters, it is difficult to distinguish berthing, anchorage anchoring, low-speed sailing or ship waiting behavior outside the port. In addition, although some scholars have defined the ship waiting speed based on experience and specifically identified the ship waiting behavior outside the port^24–26^, due to the impact of wind, waves, and current on the ship waiting speed and the problems of AIS equipment, the ship waiting speed may be close to or exceed the low-speed sailing speed. It means that using the estimated speed threshold is likely to identify the ship waiting outside the port incorrectly. Therefore, firstly, based on the AIS data of ships, this paper determines the speed threshold of ships waiting outside the port by analyzing the characteristics of ship speed distribution; secondly, the range of ships berthing in the port is distinguished, and the different waiting behavior of ships are clarified; finally, the situation of different types and sizes of ships waiting outside the port is analyzed. It is significant to strengthen the management of port traffic organization and optimize the ship voyage plan.

The main content framework of this paper is as follows: In Section "Data description and processing", the AIS data of the ship is cleaned based on the change in ship speed and course; in Section "Identification method of the ship waiting behavior outside the port", ship waiting behavior is identified through three dimensions: ship waiting speed threshold, ship waiting waters and ship waiting time; in Section "Analysis of the results of the ship waiting behavior outside the port", the behavioral characteristics of ships waiting outside the port were analyzed; discussion, conclusions and future research areas are included in Sections "Discussion" and "Conclusion".

## Data description and processing

### Ship AIS data

Ship AIS is a spatiotemporal data recording tool for ships engaged in various water activities. AIS data contains rich knowledge of ship behavior and is the primary data source for excavating ship activity patterns^27^. Ship AIS can automatically provide dynamic information such as time stamp, latitude and longitude, heading, course over ground, and speed corresponding to each position point of the ship, and other static information such as ship maritime mobile service identity (MMSI) number and ship name. It can manually input and update the sailing status, ship draught, destination port, and other voyage-related information^28^. However, AIS data has certain unreliability. Due to the performance characteristics of ship AIS equipment and the complexity of ship navigation environment, AIS data is often lost or erroneous under the interference of obstacles or signal transmission^29^. In addition, navigation and positioning systems such as the global positioning system (GPS) provide the position and other information in the AIS data. Due to the drift phenomenon^30^ of GPS and other problems, the acquired ship position, speed, course, and other information may also be wrong.

### AIS data selection

According to the International Convention for Safety of Life at Sea (SOLAS) requirements, at the end of 2004, all international voyage ships of over 300 gross tonnages, coastal navigation ships of over 500 gross tonnages, and all passenger ships must be required to install AIS equipment. As passenger ships, fishing boats, engineering ships, and other types of ships generally do not have waiting behavior outside the port, the objects studied in this paper are mainly commercial cargo ships such as tankers, bulk cargo ships, and container ships.

**Figure 1:**
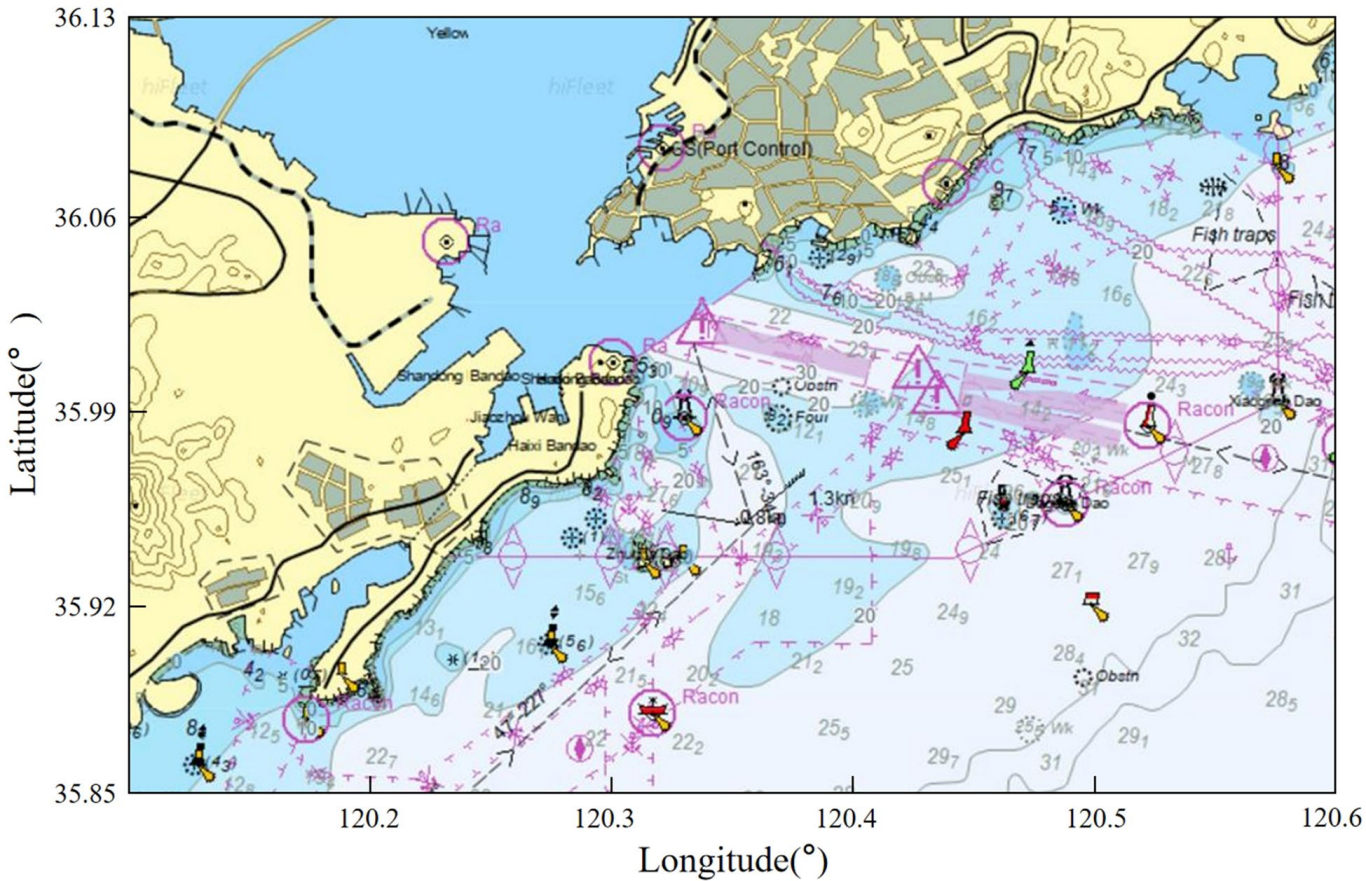
The chart of the research scope of Qingdao port waters.

The selected port waters object is the AIS data of ships in the waters near Qingdao Port, China, in March 2022. The specific water area is 120.10°–120.60° longitude and 35.85°–36.13° latitude, as shown in Fig. 1, taken from Hifleet^31^.

The data fields include static information such as ship MMIS number, ship name, ship type, length and breadth, dynamic information such as time stamp, latitude and longitude, speed over the ground and course, and navigation status information such as ship sailing status. Examples of data are shown in Table 1.

**Table 1:** Selected AIS data Sample.

MMSI	Time	Longitude	Latitude	Course	Speed	Navigation status	Ship type	Length	Breadth	Ship Name
353686000	2022/3/31 10:48:03	120.7444	35.0202	101	13.2	Undefined	Bulk cargo ship	210	32	EAGLE EXPRESS
353686000	2022/3/31 10:49:42	120.7518	35.0191	100	13.1	Undefined	Bulk cargo ship	210	32	EAGLE EXPRESS
353,686,000	2022/3/31 10:57:32	120.7858	35.0135	104	13.1	Undefined	Bulk cargo ship	210	32	EAGLE EXPRESS
477184900	2022/3/17 12:48:19	120.8435	35.3118	258.4	10.5	Underway	Tanker	333	60	DHT BRONCO
477184900	2022/3/17 12:51:48	120.8313	35.3097	257.8	10.6	Underway	Tanker	333	60	DHT BRONCO
477184900	2022/3/17 13:05:58	120.7813	35.3008	258.1	10.8	Underway	Tanker	333	60	DHT BRONCO

### AIS data processing

Due to the abnormity and unreliability of AIS data, it is necessary to preprocess the AIS data to ensure the accuracy of the research results. According to the research needs, ship speed and course changes are considered in the critical AIS data. Since the ship AIS sends a signal every 2 s to 3 min, in order to remove unimportant data, this paper selects the ship AIS data containing 100 or more location points or a sailing time of more than 15 min for analysis. To remove abnormal points and repeated values, Qu et al. adopted the method of preprocessing the AIS data by the average speed, added the change of the ship course angle, regarded the AIS data set of each ship as a continuous speed and course space–time sequence, analyzed the abnormal fluctuation of the space–time sequence in combination with the ship navigation and stay characteristics, and completed the cleaning of the selected data^32^.

Firstly, average speed ${\overline{\text{v}}}_{{{\text{i}},{\text{t}}_{{\text{j}}} }}$ and course angle change $\Delta \psi_{i}$ at each ship location point are calculated using the following equations.

1 $$\overline{v}_{{i,t_{j} }} = \frac{{\sqrt {(x_{{i,t_{j + 1} }} - x_{{i,t_{j} }} )^{2} - (y_{{i,t_{j + 1} }} - y_{{i,t_{j} }} )^{2} } }}{{t_{j + 1} - t_{j} }}$$

2 $$\Delta \psi_{{i,t_{j} }} = \psi_{{i,t_{j + 1} }} - \psi_{{i,t_{j - 1} }}$$

In Eq. (1), ${\overline{\text{v}}}_{{{\text{i}},{\text{t}}_{{\text{j}}} }}$ is the average speed of the ship *i* in a specific time interval [*t*_*j*_, *t*_*j*+*1*_] (speed unit kn, time unit h), ${\text{x}}_{{{\text{i}},{\text{t}}_{{\text{j}}} }}$ is the longitude coordinate of the ship *i* at time *t*_*j*_ (unit "),${\text{y}}_{{{\text{i}},{\text{t}}_{{\text{j}}} }}$ is the latitude coordinate of the ship *i* at time *t*_*j*_ (unit "). In Eq. (2),$\Delta \psi_{\iota }$ is the course angle change of ship *i* at time *t*_*j*_, and $\psi_{{i,t_{j} }}$ is the course of the ship *i* at time *t*_*j*_.

**Figure 2:**
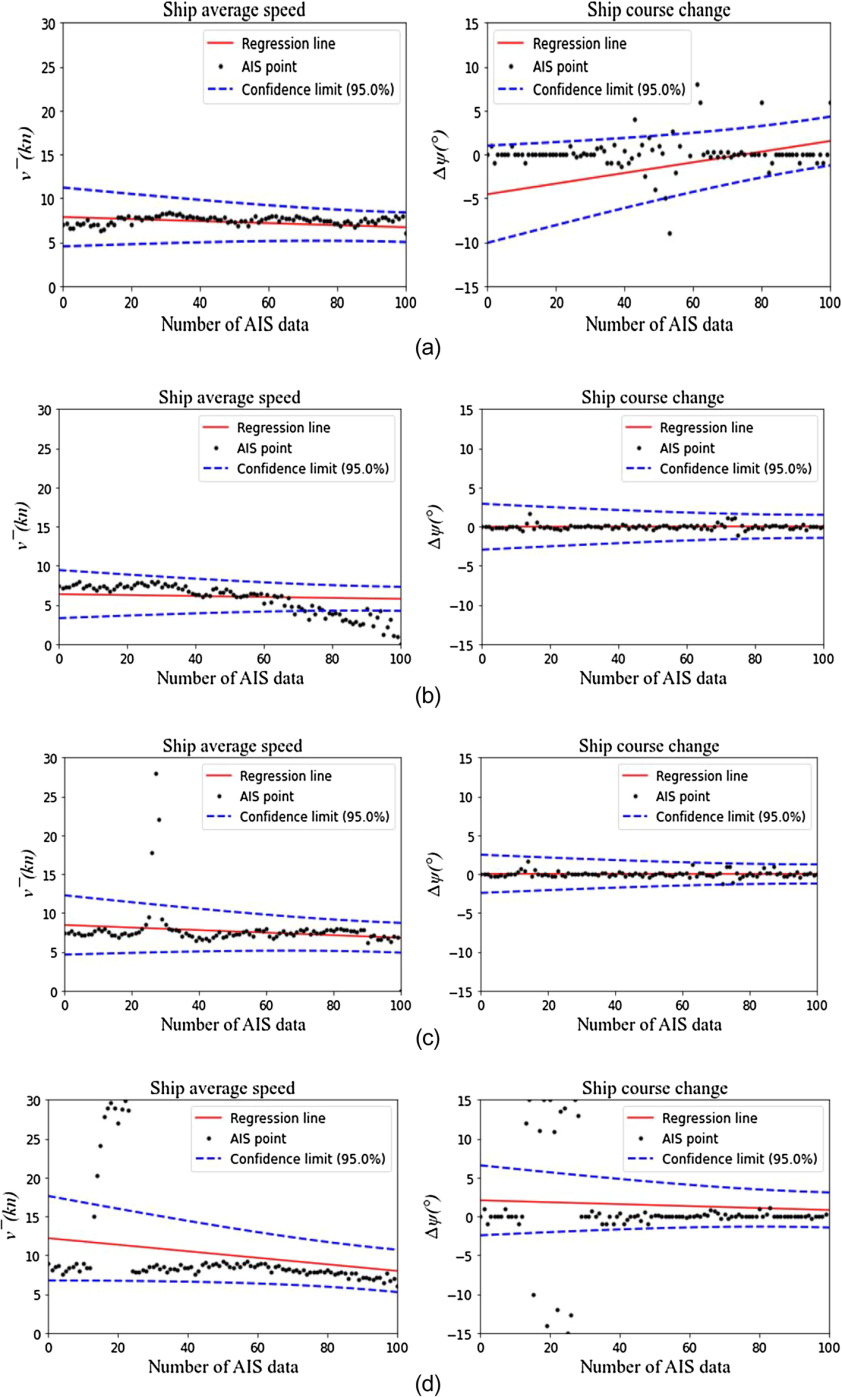
Analysis of the ship AIS data.

Furthermore, two space–time sequence anomalies of ship speed change and course change composed of ship AIS data-position points are analyzed. The augmented Dickey-Fuller (ADF) test method^33^ is used to analyze the stability of each ship track speed and course changes. For unstable ship trajectories, the 3-sigma rule^34^ is used to identify the abnormal values of speed and course changes, and analyze the abnormal behavior to identify better the ship waiting behavior outside the port. Four common situations of ship speed and course changes are shown in Fig. 2a–d.

The results of Fig. 2 are analyzed according to the ship navigation behavior. In Fig. 2a, the ship speed changes smoothly, and the course changes fluctuate. It is considered that the ship is in the normal drift state of turning around, avoiding or anchoring. In Fig. 2b, the ship speed at many continuous positions is unstable, and the course changes smoothly, which means that the ship is entering and leaving the port. In Fig. 2c, the ship speed at some positions has an abnormal value, and the course changes smoothly. It is considered that the abnormal value data of this part is incorrect. In Fig. 2d, there are multiple abnormal points in the ship speed and course changes. It is considered that the abnormal value data of this part is incorrect.

Finally, the AIS data of 501 ships in Qingdao Port and nearby waters in March 2022 is obtained to analyze ship port behavior, including 263 bulk cargo ships, 141 container ships, and 97 tankers.

## Identification method of the ship waiting behavior outside the port

**Figure 3:**
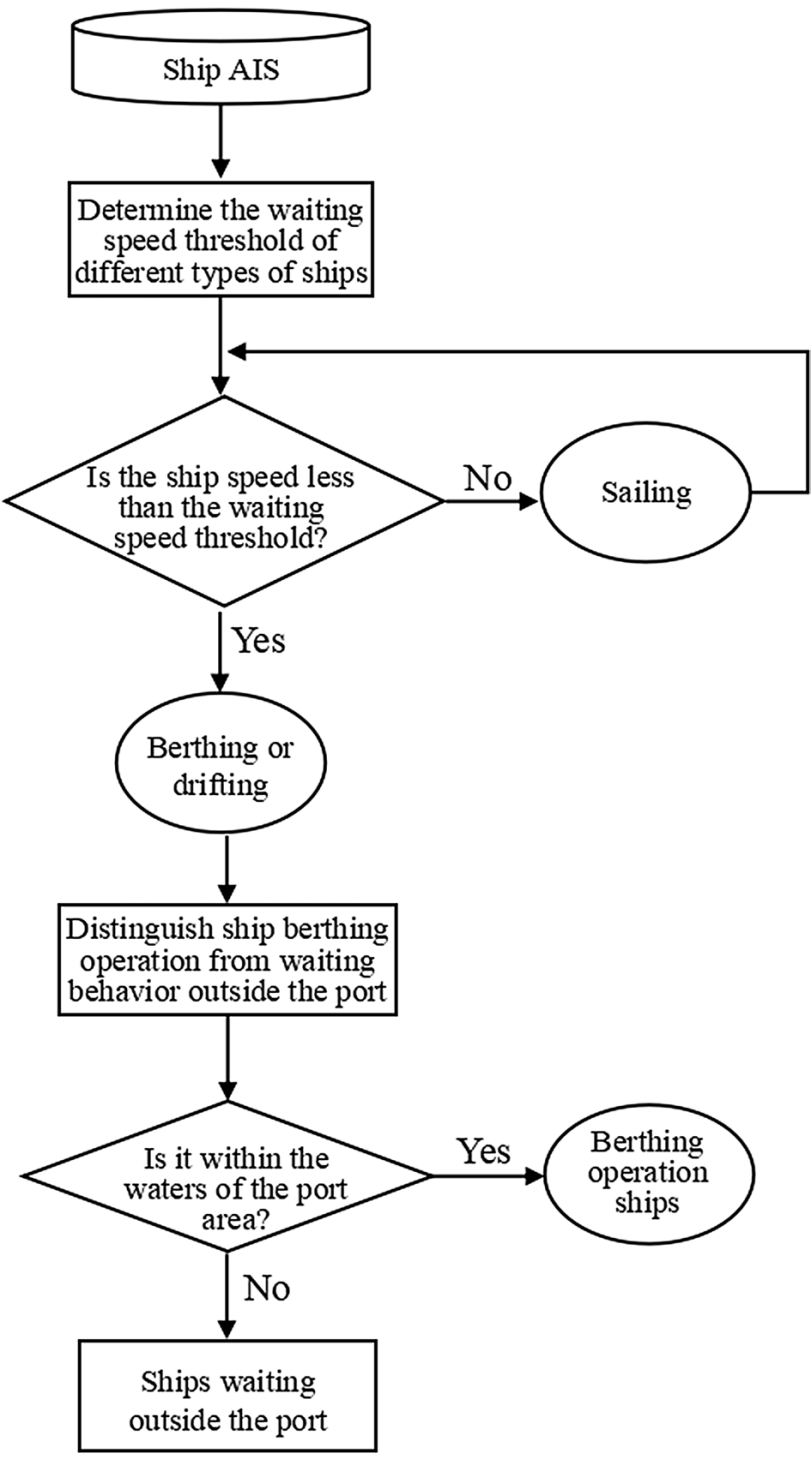
Flow chart of ship waiting behavior identification.

The specific process of the identification method of ship waiting behavior outside the port based on ship AIS data proposed in this paper is illustrated in Fig. 3.

Firstly, based on AIS data, this paper determines the waiting speed thresholds for different types of ships.When the speed of the ship exceeds the speed threshold of waiting outside the port, it is considered that the ship is in a navigation state; when the speed of the ship is less than the speed threshold of waiting outsid the port, it is considered that the ship is in a berthing or drifting state. The target ship is a ship that is in a berthing or drifting state. Then, the target ship is judged based on whether it is within the port area to distinguish between ship berthing operations behavior and waiting behavior outside the port. When the target ship is within the port area, it is considered as a berthing operation ship; when the target ship is outside the port area, it is considered a ship waiting outside the port.

### Speed threshold of ships waiting outside the port

The ship speed in the ship AIS data is an instantaneous value, which is discrete and has a certain error. At the same time, due to the effect of wind, waves, and currents, the ship is rarely in a state of zero speed while waiting outside the port. Therefore, it is the key to identifying the ship waiting behavior to determine a reasonable speed threshold of the ship waiting state outside the port.

As shown in Table 2, AIS data shows that the ship speed is greater than 0 over a long time, but the longitude and latitude of the ship are basically unchanged, and the course is unstable. Based on the analysis of the ship course behavior, the ship may be in the anchored condition, but due to the influence of external factors, the ship speed is not 0 and the course is unstable. Therefore, it is not accurate to judge the ship state only from the instantaneous speed of the ship AIS. Using the average speed of ship movement in a short time is more accurate in judging the ship running state.

**Table 2:** Examples of abnormal situations in ship AIS data.

MMSI	Time	Longitude (°)	Latitude (°)	Course (°)	Speed (kn)
413903771	0:33:33	113.2335	21.8948	125.3	2.6
413903771	0:39:32	113.2335	21.8948	125.3	1.8
413903771	3:21:38	113.2334	21.8947	125.3	2.5
413903771	4:15:39	113.2335	21.8947	125.3	2.4
…	…	…	…	…	…
413903771	19:40:41	113.2335	21.8947	186.8	3.5
413903771	20:58:14	113.2335	21.8948	213.3	1.6

**Figure 4:**
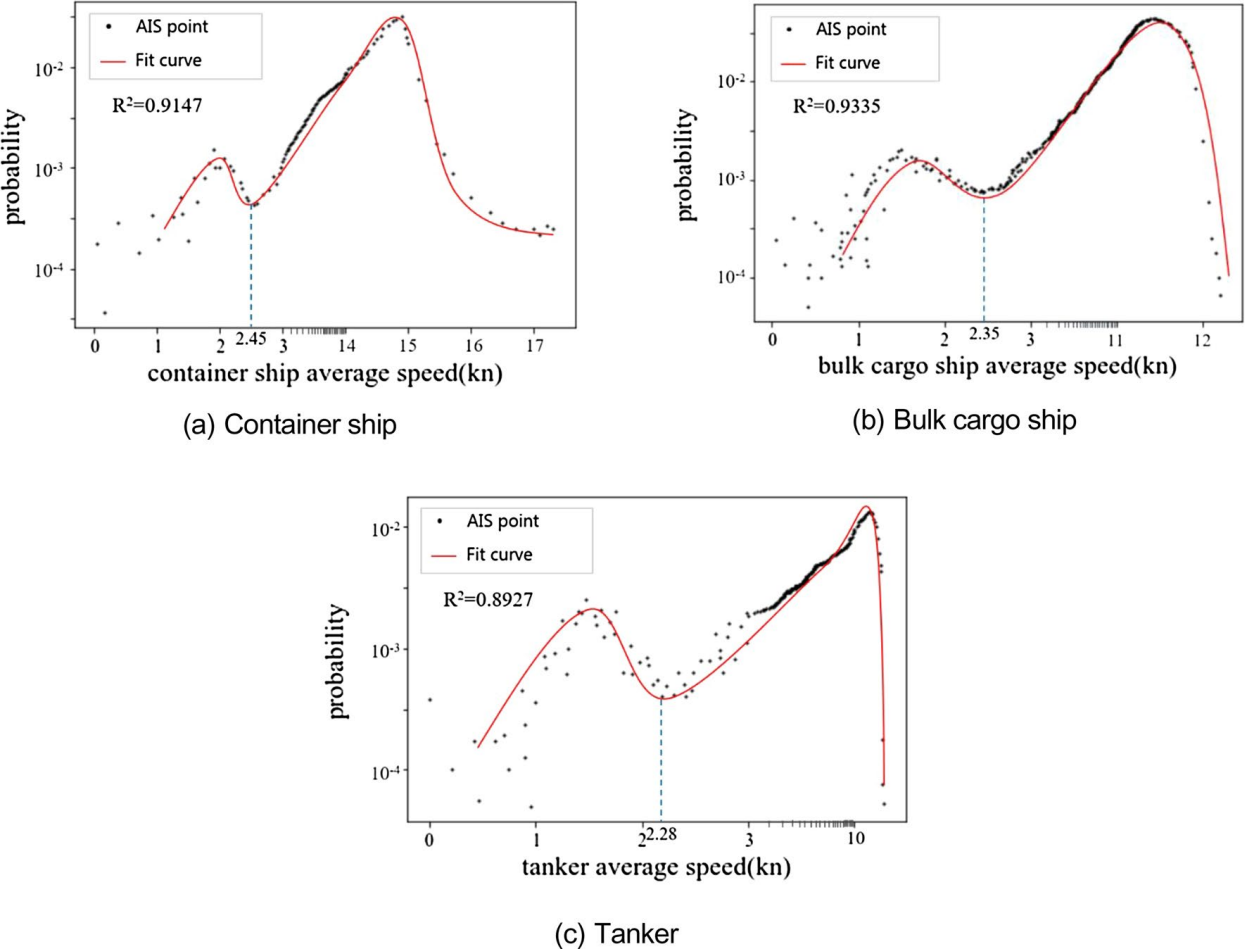
Discrete distribution and change curve of the ship average speed.

In order to determine the speed of ships waiting outside the port more accurately, this paper proposes a method to determine the speed threshold of ships waiting outside the port based on AIS data. Firstly, the cleaned ship AIS trajectory data is extracted according to the ship motion characteristics. The average speed of continuous position points is calculated according to Eq. (1). Moreover, we obtain the discrete distribution diagram of the average velocity of three common types of ships in Qingdao port waters: container ships, bulk cargo ships, and tankers, as shown in Fig. 4a–c. It can be seen that the average speed has prominent peaks and bottoms, the distribution of the higher part of the average speed tends to be smooth, while the lower part fluctuates significantly due to external factors, and there is no apparent law.

Secondly, based on the characteristics of the discrete distribution diagram of average speed, the normal distribution function shown in Eq. (3) is used to mix and fit the average speed data of three types of ships. The average speed change curve of ships in Fig. 4 is obtained.

3 $$f\left( {\overline{v}} \right) = \frac{1}{{\sqrt {2\pi } \sigma }}\exp \left( { - \frac{{(\overline{v} - \mu )^{2} }}{{2\sigma^{2} }}} \right)$$

In Eq. (3), $f\left( {\overline{v}} \right)$ is the probability distribution function of average velocity, $\sigma$ and $\mu$ are the parameters of the distribution function^35^.

Finally, it can be seen from the average speed fitting curve in Fig. 4 that there are two valley peaks in the curve, and there is a saddle point between the valley peaks, which is the local minimum value of the average speed. Here, it is determined as the upper limit speed threshold when the ship is waiting outside the port. Moreover, it is used to determine whether the ship has stopped sailing for a while.

It can be determined from Fig. 4 that the waiting speed thresholds of container ships, bulk cargo ships, and tankers are 2.45 kn, 2.35 kn, and 2.28 kn, respectively.

### Water area of ships waiting outside the port

**Figure 5:**
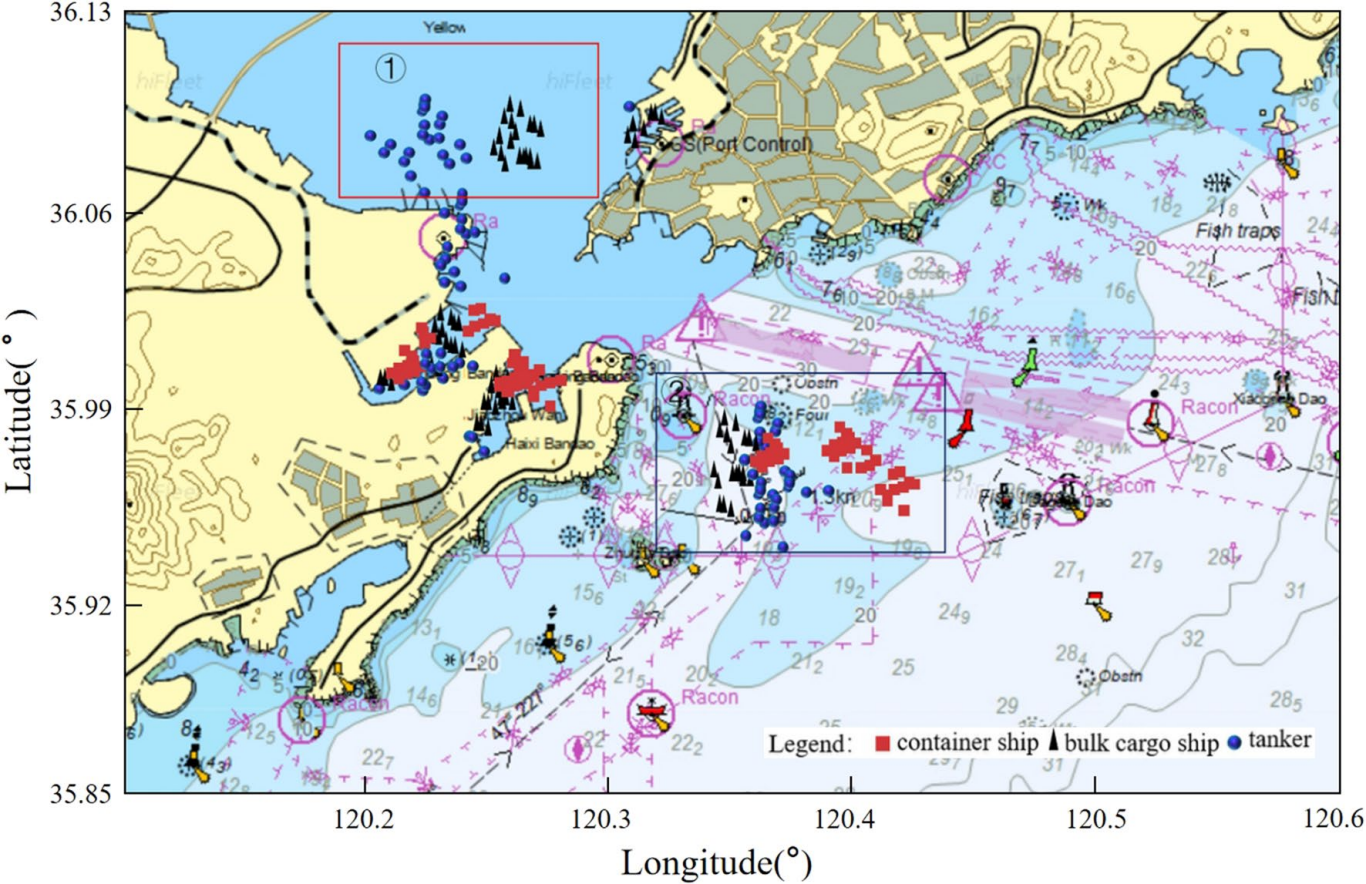
Distribution diagram of ship waiting waters outside Qingdao port.

After the speed threshold of ships waiting outside the port is determined, the distribution and number of ships whose speed in Qingdao port waters is lower than the speed threshold can be obtained. This paper uses Hifleet^31^ and Python 3.10 to draw images, as shown in Fig. 5. Since the behavior characteristics of ships when berthing and anchoring in the port are similar to those of waiting outside the port, to better distinguish the berthing behavior and waiting behavior in the port waters, the longitude and latitude of each berth of Qingdao port are extracted, and the berthing ships and anchoring ships within the port are excluded. And then, we obtained the waiting waters of ships outside Qingdao port, as shown in boxes ① and ② in Fig. 5.

It can be seen from Fig. 5 that different types of ships, such as container ships, bulk cargo ships and tankers, show obvious aggregation characteristics during the waiting period in the waters outside the port.

### Ship waiting time outside the port

**Figure 6:**
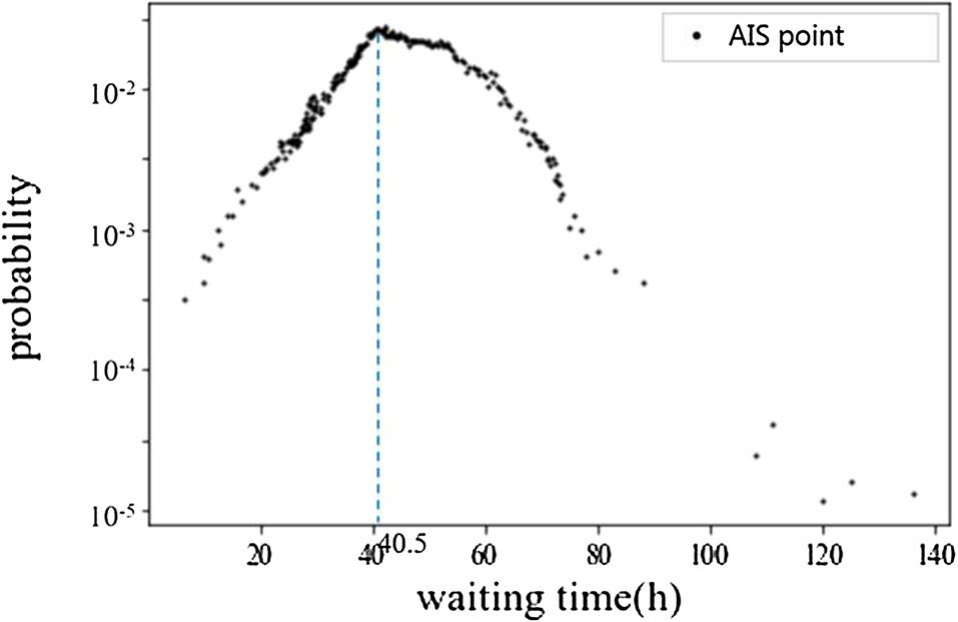
Overall probability distribution of ship waiting time.

**Figure 7:**
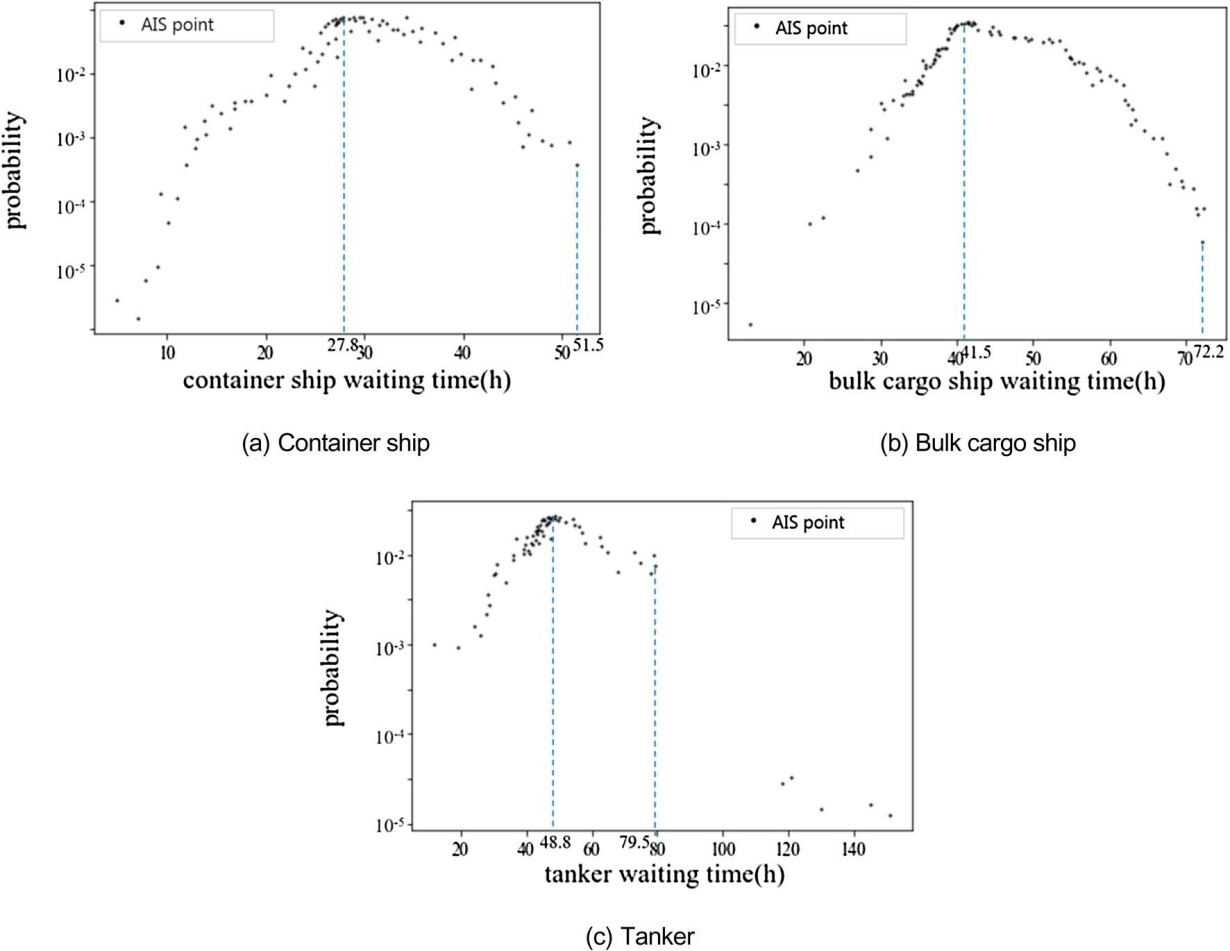
Probability distribution of waiting time of different types of ships.

In order to better analyze the behavior of ships waiting outside the port, the waiting time of ships whose average speed is lower than the calculated waiting speed threshold is statistically analyzed. Then, we obtained the overall probability distribution of ship waiting time, as well as the probability distribution of waiting time of different types of ships waiting outside the port and in the port, as shown in Figs. 6 and 7.

As can be seen from the figure above, although the waiting time outside and inside the port is affected by various factors, such as port organization, the ship itself and the navigation environment, the overall distribution shows a certain regularity, and the probability of waiting time has an obvious peak value. The distribution characteristics of the ship waiting time reflect different ship waiting behavior, that is, the operation behavior in the port and the waiting behavior outside the port.

Figure 6 shows that two common waiting behavior of ships are divided into two distribution segments by the peak value of the probability distribution of the waiting time. The peak value of the probability distribution of the waiting time is 40.5 h, and the tail value of the probability distribution of the waiting time is 80 h. That is, the average time of ships operating in the port is within 40.5 h, and the average time of ships waiting outside the port is within 80 h.

Figure 7 shows the probability distribution of waiting time of different types of ships. The distribution of the waiting time of different types of ships is different. In Fig. 7a, the waiting time of the container ship's harbour operation is 27.8 h, and the waiting time outside the port is 51.5 h. In Fig. 7b, the waiting time of the bulk cargo ship's harbour operation is 41.5 h, and the waiting time outside the port is 72.2 h. In Fig. 7c, the waiting time of the tanker's harbour operation is 48.8 h, and the waiting time outside the port is 79.5 h. In addition, a small number of tankers have been suspended for a long time, and the waiting time is more than 100 h. They may be in a short-term suspension of operation due to market reasons such as oil prices or maintenance, and this kind of behavior is not listed as the behavior of ships waiting outside the port.

Through the definition of speed threshold, water area and ship waiting time outside the port, the identification of the behavior of ships waiting outside the port is completed. In the end, the total number of ships waiting outside Qingdao port in March 2022 is 118, including 32 container ships, 51 bulk cargo ships, and 35 tankers. Among the 118 ships identified by this method in waiting outside the port, the ship sailing status recorded by the AIS raw data shows that 98 ships are anchored, moored or stopped, 5 ships are displayed as sailing, and 15 ships are in an unknown state. Therefore, judging the sailing state of ships by AIS data has a significant error, especially for determining the state of non-navigating ships.

## Analysis of the results of the ship waiting behavior outside the port

**Figure 8:**
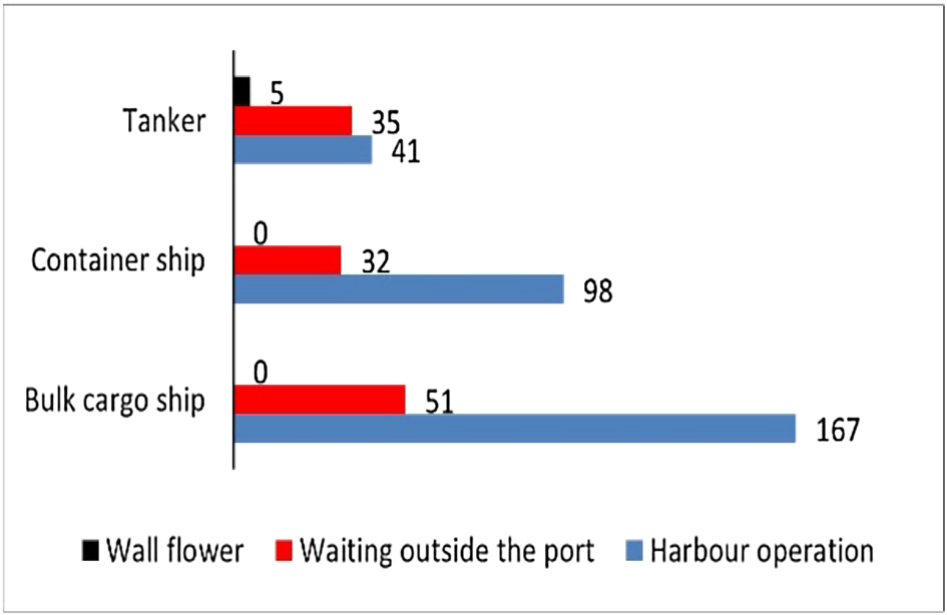
Number distribution of different types of ship status in Qingdao Port waters in March 2022.

**Figure 9:**
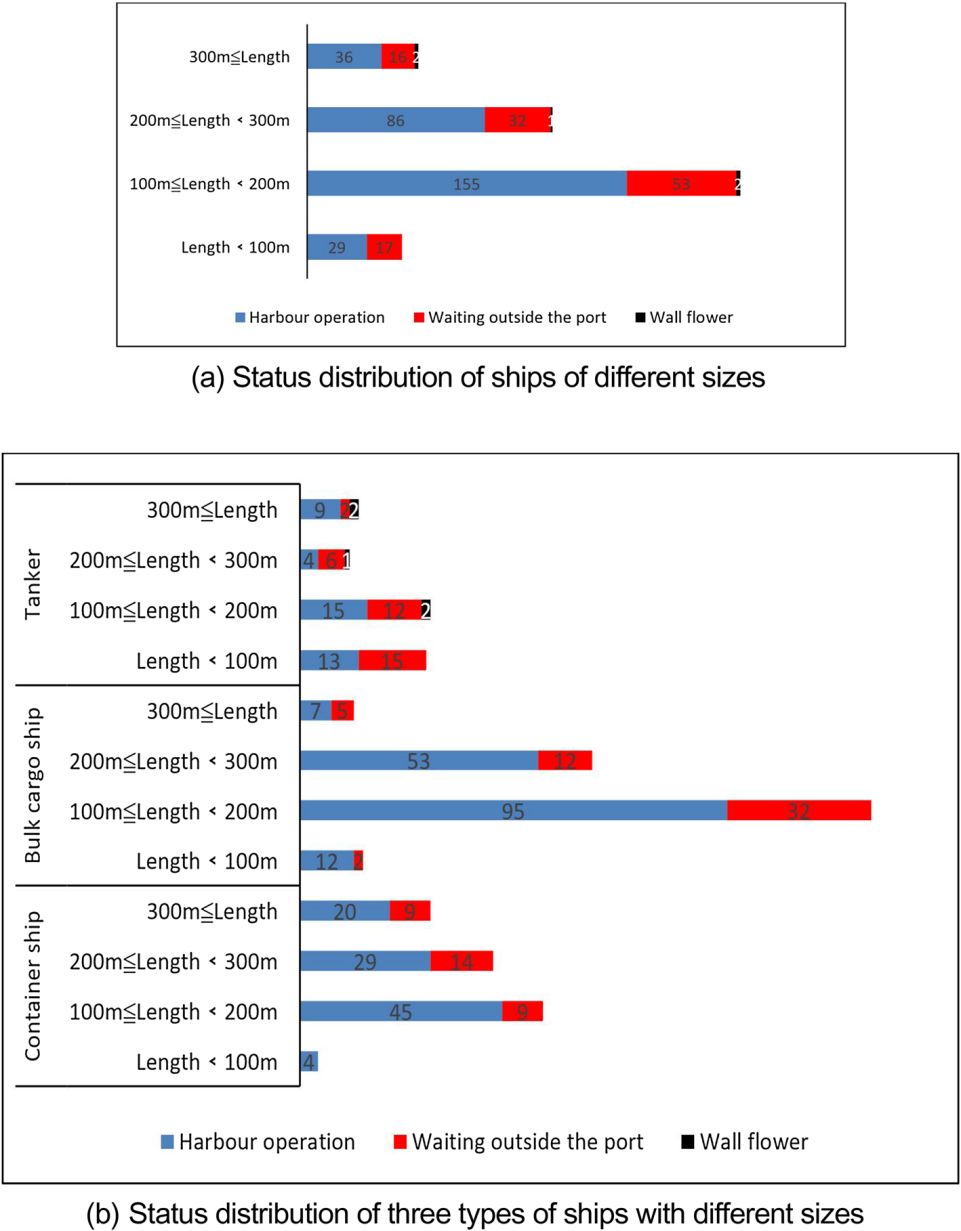
Number distribution of different sizes of ship status in Qingdao Port waters in March 2022.

There are 501 ships in the water area of Qingdao port that meet the research conditions in this study. Through the excavation and judgment of the ship waiting behavior outside the port, the distribution of the number of ships in the water area of Qingdao port in March 2022 in different status, such as sailing, berthing in the port, waiting outside the port, and long-term berthing, is shown in Figs. 8 and 9.

The results in Fig. 8 show that in March 2022, 118 ships experienced waiting behavior outside the port, accounting for 23.6% of the total number of ships in the water area, showing the distribution of ships waiting outside the port in terms of ship types. The number of bulk cargo ships in waiting status outside the port is the largest, followed by tankers and container ships. However, the proportion of tankers in waiting status outside the port is the highest at 36%, and the proportion of container ships and bulk cargo ships in waiting status outside the port is 22.7% and 19.4%, respectively.

The results in Fig. 9a show that, in terms of ship size, the largest number of ships waiting outside the port is those with a length of 100 m–200 m, 53 ships, accounting for 45% of the total number of ships waiting outside the port; followed by those with a length of 200 m–300 m, 32 ships, accounting for 27% of the total number of ships waiting outside the port. This corresponds to the overall number distribution of ship size.

The results in Fig. 9b show the number distribution of container ships, bulk cargo ships, and tankers at different sizes in waiting status outside the port. The container ships with the largest number waiting outside the port are those with a length of 200 m–300 m, the bulk cargo ships with the largest number waiting outside the port are those with a length of 100 m–200 m, and the tankers with the largest number waiting outside the port are those with a length of less than 100 m. This may be related to tankers' special loading and unloading operation process.

According to the analysis results in Fig. 7, the waiting time of different types of ships outside the port is shown in Table 3. The waiting time of different sizes of ships outside the port is shown in Table 4. It can be observed that the longer the ship, the longer its waiting time at the port is basically also. The number of ships less than 200 m in length is higher, but the overall waiting time is shorter. The number of ships longer than 200 m is smaller and decreases as the ship’s length increases, but the average waiting time is significantly longer.

**Table 3:** The waiting time of different types of ships outside the port.

Ship types	The number of ships waiting outside the port	Average waiting time (h)
Container ship	32	51.5
Bulk cargo ship	51	72.2
Tanker	35	79.5

**Table 4:** The Waiting time of different sizes of ships outside the port.

Ship sizes	The number of ships waiting outside the port	Average waiting time (h)
Length < 100 m	17	39.5
100 m ≦ Length < 200 m	53	38.2
200 m ≦ Length < 300 m	32	48.8
300 m ≦ Length	16	54.6

## Discussion

In this paper, the ship navigation behavior is analyzed with the aid of ship AIS data. Taking the sea area of Qingdao Port as an example, the ships in the sea area are analyzed according to the behavior of ships underway, berthing in the port, waiting outside the port, and long-term berthing, and the characteristics of ships waiting outside the port are emphatically identified. From the analysis of the above identification results, due to the impact of external uncontrollable factors on the marine transportation industry, ships of different types and sizes have distinctive clustered waiting behavior outside the port, and there are significant differences in waiting time. Among the ship types, the number of bulk cargo ships waiting outside the port is the largest, followed by tankers, which is consistent with the overall distribution of bulk cargo ships. The low handling efficiency is also the main reason for the large number and long waiting time of bulk cargo ships waiting outside the port. By comparison, container ships spend the least time waiting outside the port. From the comparison of the waiting time of container ships in Qingdao Port in the past, it can be seen that the overall operational efficiency of container ships in Qingdao Port has a relatively small impact when global port congestion is relatively common, which is related to the efficient operational efficiency and automation of container ships and Qingdao Port.

In terms of ship size, the number of ships waiting outside the port decreases with the increase in ship size, but the average waiting time is significantly longer. The ship size greatly impacts the efficiency of entering and leaving the port. At the same time, the loading, unloading, and berth-shifting operation efficiency of large ships in the port is relatively low, which also leads to frequent congestion of large ships.

The ship waiting outside the port not only increase the degree of maritime traffic congestion, but also have many uncertain factors affecting maritime traffic safety. Therefore, the port authority can reasonably organize ships to enter and leave the port according to the characteristics and actual conditions of the ship waiting behavior of different types and sizes outside the port. Shipping companies can also select appropriate ship types and plan routes according to different port congestion.

This paper only selects the ship waiting behavior outside the Qingdao Port in March 2022 for identification and analysis. If cross year ship AIS data are selected for comparative analysis, it will better illustrate the impact of external factors on port congestion. At the same time, the analysis results of ship AIS data with a large time span may be more valuable for research. In addition, the method proposed in this paper for mining the ship waiting behavior outside the port can also be applied to other ports to identify and analyze the ship waiting behavior outside the port, helping to solve port congestion problems and achieve optimal route planning.

## Conclusion

Accurately identifying the behavior of ships waiting outside the port can optimize the port organization and management plan to reduce port congestion. And it is crucial for ships to reasonably plan their navigation, master the congested area and waiting time, and reduce operating costs. For this reason, this paper excavates and analyzes the behavior of ships waiting outside the port based on ship AIS data. Firstly, we preprocess the collected AIS data of the ship, and use the ADF inspection method to analyze the stability of the ship speed and course change sequence, so as to clarify the ship normal navigation behavior and abnormal conditions. Secondly, considering the abnormal and unstable situation of AIS data, to more reasonably determine the waiting behavior of ships outside the port, we use the average speed of ships to determine the waiting speed threshold of container ships, bulk cargo ships, and tankers. Thirdly, we distinguish the berthing area in the port and the waiting area outside the port. And we focus on analyzing the time variation characteristics of ship waiting behavior. The results show that the time of the whole waiting activity of the ship has a prominent peak. The peak divides the whole waiting behavior of the ship into different stages, which can better reflect the different waiting behavior of the ship. Finally, we analyze the number and waiting time of different types and sizes of ships waiting outside the port. The research results indicate that due to uncontrollable factors, ships of different types and sizes exhibit distinctive clustered waiting behavior outside the port, and there are significant differences in waiting time. From the perspective of ship types, bulk cargo ship have the highest number among ships waiting outside the port, followed by tanker and container ship; from the perspective of ship scale, basically, the number of ships waiting outside the port decreases with the increase of ship scale, but the average waiting time is longer. The port authority can reasonably organize ships to enter and leave the port according to the characteristics and actual conditions of the ship waiting behavior of different types and sizes outside the port, to ensure smooth and safe navigation of waterways; shipping companies can better plan routes based on these behavioral characteristics to avoid congestion. The research results have certain application value for improving port operation efficiency and accelerating ship turnover speed.

Although this study reveals some important findings regarding ship waiting behavior, there are still some shortcomings. Firstly, this paper only selects the month in which the COVID-19 epidemic broke out in Qingdao in March 2022 to identify and analyze the ship waiting behavior outside the port. If the annual ship AIS data before and after the COVID-19 epidemic broke out are selected for comparative analysis, it will better illustrate the impact of the epidemic and other external factors on port congestion. Secondly, selecting AIS data analysis results for ships with a larger time span may have more research value. Finally, this paper only identifies ship waiting behavior outside the port. If further research is conducted on the causes of ship waiting behavior and the classification of ship waiting behavior, more accurate suggestions will be provided to solve the problem of port congestion.

## Data Availability

All data generated or analysed during this study are included in this published article.
